# Green carbon dots derived from *Zingiberis Rhizoma Carbonisatum* alleviate ovalbumin-induced allergic rhinitis

**DOI:** 10.3389/fimmu.2024.1492181

**Published:** 2024-11-28

**Authors:** Long Zhou, Yingxin Yang, Tingjie Li, Yafang Zhao, Jinye Yuan, Chenxin He, Yan Huang, Jinyu Ma, Yue Zhang, Fang Lu, Jiaze Wu, Zijian Li, Hui Kong, Yan Zhao, Huihua Qu

**Affiliations:** ^1^ School of Traditional Chinese Medicine, Beijing University of Chinese Medicine, Beijing, China; ^2^ School of Chinese Materia Medica, Beijing University of Chinese Medicine, Beijing, China; ^3^ School of Life Sciences, Beijing University of Chinese Medicine, Beijing, China; ^4^ Center of Scientific Experiment, Beijing University of Chinese Medicine, Beijing, China

**Keywords:** carbon dots, herbal medicine, *Zingiberis Rhizoma Carbonisatum*, allergic rhinitis, inflammation, metabolism

## Abstract

**Background:**

Allergic rhinitis (AR) affects up to 40% of the population, leading to significant healthcare expenditures. Current mainstream treatments, while effective, can lead to side effects and do not address the underlying immunological imbalances. *Zingiberis Rhizoma Carbonisatum* (ZRC), the partially charred product of *Zingiberis Rhizoma* (ZR), has been widely used clinically in China since ancient times to treat respiratory disorders.

**Methods:**

Inspired by the similarity between high-temperature pyrolysis and carbonization processing of herbal medicine, ZRC derived CDs (ZRC-CDs) were extracted and purified through several procedures. Then, the physicochemical characteristics of CDs were delineated through a suite of characterization methods. Moreover, our investigation zeroed in on elucidating the ameliorative impacts of CDs on ovalbumin-induced rat models alongside their underlying mechanisms.

**Results:**

ZRC-CDs with particle sizes ranging from 1.0 to 3.5 nm and rich surface functional groups. Additionally, we observed that ZRC-CDs significantly attenuated nasal symptoms and pathological damage in ovalbumin-induced AR rats, and modulated lipid metabolism and type 2 inflammatory responses. They also inhibit PI3K/AKT and JAK/STAT pathways, which are associated with metabolism and inflammation. Importantly, ZRC-CDs demonstrated high biocompatibility, underscoring their potential as a novel therapeutic agent.

**Conclusion:**

ZRC-CDs offer a promising alternative for AR treatment and could help facilitate broader clinical use of the ZRC. In addition, the exploration of the inherent bioactivity of CDs can help to broaden their biological applications.

## 1 Introduction

Allergic rhinitis (AR), an IgE-mediated type 1 hypersensitivity reaction of the nasal mucosa, results from exposure to allergens. It manifests through symptoms such as nasal congestion, itching, a runny nose, and sneezing (1). The immunological basis of AR is primarily a type 2 inflammatory response (2–4). Additionally, there is growing evidence suggesting a significant role of metabolic disorders in the development of allergic diseases. In recent years, the prevalence of AR has steadily increased to between 5% and 40%, imposing a substantial medical and economic burden (5). Beyond diminishing quality of life, AR is also correlated with several comorbidities including asthma, anxiety, and depression (6–8). Current pharmacological treatments for allergic rhinitis, such as H1 receptor antagonists, corticosteroids, and leukotriene receptor antagonists, often lead to side effects and financial strains over long-term use, without addressing the underlying immune imbalance (6). Meanwhile, allergen immunotherapy (AIT) is becoming more widely recognized; however, it presents risks of serious adverse effects and generally suffers from poor patient compliance (5). Therefore, there is an urgent need to propose new solution strategies for AR.

As a 0-dimensional nanomaterial, carbon dots (CDs) exhibit several unique advantages, including ultra-small size, tunable photoluminescence, high photostability, low toxicity, easy surface modification, and excellent aqueous dispersibility (9–15). These characteristics not only make CDs versatile but also highly applicable in biomedical contexts. Particularly noteworthy are the bioactivities of biomass-derived CDs, which include anti-inflammatory effects, immune modulation, lipid metabolism regulation, antioxidative stress capabilities, and anti-allergic properties (16–23). Additionally, in comparison with chemically synthesized CDs, biomass-derived CDs possess several benefits, such as lower production costs, less complex preparation methods, and improved biocompatibility (21, 23–25). Collectively, these attributes underscore the potential of CDs as an innovative treatment strategy for allergic rhinitis.

*Zingiber offcinale Rosc.* is a plant that widely used as a flavor and dietary supplement. The dried rhizome of *Zingiber offcinale Rosc.*, known as *Zingiberis Rhizoma* (ZR), has been used as an Chinese materia medica for thousands of years. The ‘Treatise on Cold Damage and Miscellaneous Diseases’, revered as the foundational text of herbal formulations, notes that partially charred product of ZR as a treatment for respiratory ailments. Furthermore, recent studies corroborate that ZR and its extracts are effective against various respiratory disorders, including AR and asthma (26–28). Interestingly, our research has identified CDs in commercially available *Zingiberis Rhizoma Carbonisatum* (ZRC, obtained from ZR by high-temperature stir-frying to partially charred, Chinese Pharmacopoeia, stir-frying charcoal method 0213), which we have named ZRC-CDs. We developed a series of methods to extract, purify, and characterize these ZRC-CDs, focusing on their morphology, optical features, and surface composition. Based on all of these findings, we hypothesize that ZRC-CDs, with their electron-exchange capabilities and rich functional groups, could be effectively employed in the treatment of AR.

## 2 Materials and methods

### 2.1 Materials

Ovalbumin, OVA (A5503, Sigma-Aldrich, USA); Aluminium Hydroxide Gel Adjuvant (vac-alu-250, InvivoGen, Canada) 10 mg/mL; Loratadine (L129223-5g, Aladdin); 0.9% NaCl was purchased from Jiangsu Changjiang Pharmaceutical Co., Ltd. (Yancheng, China). Interleukin-4 (IL-4, ml102825), interleukin-5 (IL-5, ml002975), interleukin-13 (IL-13, ml003012), OVA-specific IgE (OVA-sIgE, ml003273), and histamine (HIS, ml002986) kits were purchased from Shanghai Enzyme-linked Biotechnology Co., Ltd. (Shanghai, China).

ZRC was sourced from Beijing Qiancao Herbal Pieces Co., Ltd. (Beijing, China). Each batch was quality-verified by the National Medical Products Administration of China to ensure compliance with relevant standards. All herbs purchased were authenticated by Prof. Yan Zhao from the Beijing University of Chinese Medicine. Dialysis bags with a molecular weight cut-off of 1000 Da were procured from Beijing Ruida Henghui Technology Development Co., Ltd. (Beijing, China). All other analytically-grade chemical reagents were provided by Sinopharm Chemical Reagent Beijing Co., Ltd. (Beijing, China). Deionized water (DW) or ultrapure water (UPW) was used in all experiments.

### 2.2 Animals

Six-week-old male SD rats, SPF grade, weighing approximately 200 ± 20 g, were obtained from Beijing Vital River Laboratory Animal Technology Co., Ltd (Beijing, China; certificate number: SYXK (Beijing) 2020-0033). Prior to experimental procedures, the rats were acclimatized for one week in an isolation room. The environment was precisely controlled at a temperature of 23 ± 1°C and humidity of 55 ± 5%, with a 12-hour light/dark cycle. The animals had free access to food and water throughout the acclimatization and experimental periods.

The animal study was approved by the Ethics Review Committee of Animal Experimentation of the Lunan Pharmaceutical Group Co., Ltd. (Ethics Number: AN-IACUC-2023-075). The study was conducted in accordance with the local legislation and institutional requirements.

### 2.3 Preparation and characterization of ZRC-CDs

#### 2.3.1 Preparation of ZRC-CDs

ZRC was first pulverized into a fine powder using a high-speed grinder. This powder was soaked in DW and subsequently heated at 100°C in a digital thermostatic water bath (Model HH-2, Shanghai Lichen Instrument Technology Co., Ltd.) for three separate one-hour sessions to ensure thorough extraction. After heating, the mixture was filtered through a 0.22 μm nitrocellulose membrane. The filtrate was then dialyzed using dialysis membranes with a MWCO of 1000 Da. This dialysis process was conducted over a period of fifteen days to ensure the removal of small molecule compounds. The purified CDs were subsequently collected and stored at 4°C for further analysis.

#### 2.3.2 Characterization of ZRC-CDs

The morphology and microstructure of ZRC-CDs were characterized at an accelerating voltage of 200 kV using transmission electron microscopy (TEM, Tecnai G2 F20, FEI company, USA). The lattice spacing of ZRC-CDs was observed using high-resolution transmission electron microscopy (HRTEM, JEN-1230, JEOL Ltd., Japan). The optical properties of ZRC-CDs were scanned and detected by ultraviolet-visible spectroscopy spectrophotometer (UV-vis, CECIL, UK) and fluorescence spectroscopy (F-4500, Hitachi, Japan). Surface functional groups in ZRC-CDs were identified through Fourier transform infrared spectroscopy (FTIR, Thermo Fisher, USA) across a wavelength range of 400-4000 cm^-1^. Surface composition and elemental analysis of ZRC-CDs were recorded using x-ray photoelectron spectroscopy (XPS, ESCALAB 250 Xi, Thermo Fisher Scientific, USA) and excitation by a single x-ray source, Al Kl (1486.6 eV). The ZRC-CDs diffraction peaks were identified by x-ray diffraction (XRD) performed with D8-Advanced X-ray diffractometer (Bruker AXS, Germany) to elucidate their crystalline structure.

#### 2.3.3 ZRC-CDs component analysis of ZR and ZRC-CDs by HPLC method

ZR was finely ground, and 0.5 g of both ZR powder and ZRC-CDs lyophilized powder were precisely weighed and dispersed in 20 mL of methanol, followed by sonication for extraction. The resulting solutions were filtered through a 0.22μm membrane and 10 μL were injected into an Agilent 1260 series high-performance liquid chromatograph for analysis. This system includes a thermostatically controlled ZORBAX SL-C18 column (4.6 mm × 250 mm × 5 μm), an autosampler, degasser, quaternary pump, and a diode array detector. Chromatographic separation employed a mobile phase of 0.1% phosphoric acid (solvent A) and acetonitrile (solvent B), with a gradient elution of: 0-10 min, 90-75% A; 10-30 min, 75-60% A; 30-45 min, 60-43% A; 45-60 min, 43-35% A; 60-75 min, 35-20% A, at a flow rate of 1 mL/min. The column temperature was maintained at 30°C, and detection was performed at 254 nm.

### 2.4 OVA-induced AR rat model and treatments

Rats were randomly divided into six groups (n = 10): control, OVA, loratadine, low-dose ZRC-CDs (ZRC-CDs-L), medium-dose ZRC-CDs (ZRC-CDs-M), and high-dose ZRC-CDs (ZRC-CDs-H). The allergic rhinitis (AR) model was established following a modified version of a previously reported procedure (29–31). 0.2 mg OVA was dissolved in 0.5 mL saline and thoroughly mixed with 0.5 mL aluminium hydroxide gel adjuvant to form a suspension. This suspension was administered intraperitoneally to the rats every other day for a total of seven injections. Nasal challenges were conducted as follows: from days 15 to 17, rats received a 1% OVA saline solution titrated at 50 μL per nasal cavity daily; from days 18 to 19, the concentration was increased to 2.5%, administered at the same volume; and from days 20 to 29, a 5% OVA saline solution was administered daily. In the control group, both the intraperitoneal injection and nasal administration were replaced with an equivalent volume of saline.

**Figure 1:**
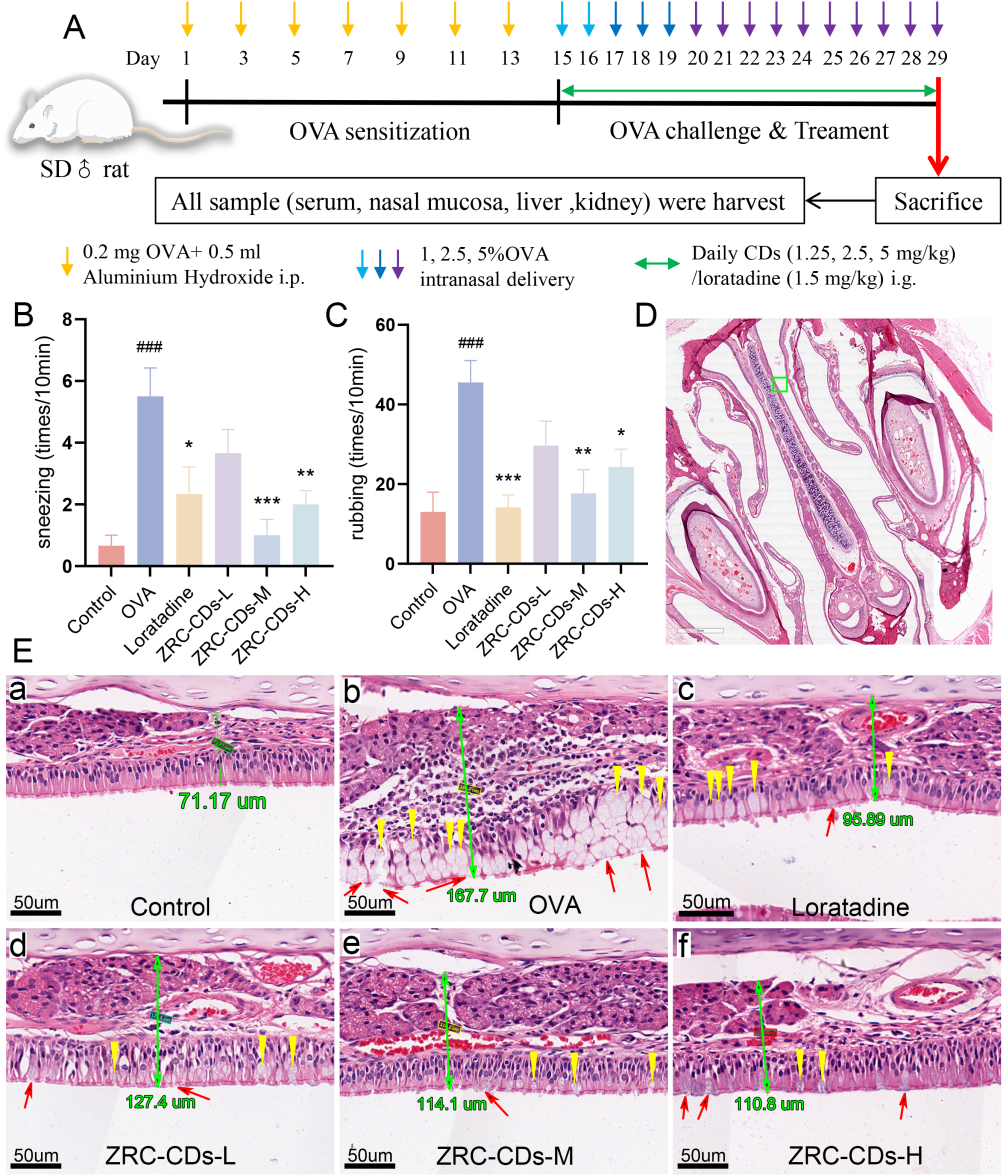
CDs alleviated OVA-induced AR. **(A)** Experimental flow chart. **(B, C)** AR Behavioral statistics: number of sneezing and rubbing in 10 minutes. Each group n = 6. **(D)** H&E staining of nasal pathology sections, scale bars: 2x. The green ‘□‘ represents magnification areas. **(E, A–F)** Respective magnified images of six groups, scale bars: 40x. The green number is the thickness of nasal mucosa; yellow ‘▽’ represent goblet cell proliferation; red ‘↑’ represent cilia disruption and discontinuity. Each group n = 3. ###*p* < 0.001 vs control group; *p < 0.05, **p < 0.01, ***p < 0.001 vs OVA group.

Besides, from days 15 to 29, each group of rats received their respective treatments as follows: the control and OVA groups (1 mL/100 g of saline); the loratadine group (1.5 mg/kg of loratadine); and the three ZRC-CDs groups (5 mg/kg, ZRC-CDs-H; 2.5 mg/kg, ZRC-CDs-M; 1.25 mg/kg, ZRC-CDs-L). All administration was via i.g. (**Figure 1A**).

### 2.5 Evaluation of nasal symptoms

Immediately after the final nasal drop challenge, the frequency of sneezing and nose scratching was recorded over a 10-minute period by an observer blinded to the group assignments of each rat.

### 2.6 Enzyme-linked immunosorbent assay

All rats were anaesthetized with zoletil 50 (6 mg/kg) and xylazine hydrochloride mixture (12 mg/kg), and blood sample were collected via the intraperitoneal vein using vacuum blood collection tubes (BD Biosciences, USA). The collected samples were then centrifuged at 3500 rpm for 10 minutes to separate the supernatant, which was carefully aspirated. THE levels of OVA-sIgE and HIS were quantified using enzyme-linked immunosorbent assay (ELISA) kits. Additionally, the levels of Th2 cytokines (IL-4, IL-5, IL-13) were detected. All procedures were meticulously executed in strict accordance with the specifications provided by the assay kits.

### 2.7 Histopathological analysis

Rat head tissues were initially fixed in a 4% paraformaldehyde solution (Sigma-Aldrich, USA) for two days, followed by decalcification in a 10% EDTA solution (Sigma-Aldrich, USA) for 21 days. After decalcification, the tissues underwent a dehydration process using a series of gradient ethanol and xylene solutions before being embedded in paraffin. The embedded tissues were then sectioned into 5-μm-thick slices and mounted on slides using neutral gum. For histological examination, the sections were stained with hematoxylin and eosin (H&E) (Sigma-Aldrich, USA). Pathological changes in the nasal mucosa were observed and analyzed under a light microscope.

### 2.8 Western blot analysis

Nasal mucosal tissues were lysed using RIPA Lysis Buffer (Beyotime Biotechnology, Shanghai, China) with the addition of protease and phosphatase inhibitors. The protein concentration in the lysates was quantified using the BCA Protein Assay Kit (Biorigin Inc., Beijing, China). Proteins (30 μg per sample) were separated by electrophoresis on either 8% or 10% SDS-PAGE gels and then transferred onto NC membranes. These membranes were blocked using a solution of either 5% skimmed milk or 5% BSA for 1 hour at room temperature. After blocking, the membranes were washed thoroughly with TBST buffer (5 min × 3 times) and incubated with primary antibodies overnight at 4°C. Following another washing step (5 min × 3 times), the membranes were incubated with a secondary antibody (1:8000, Proteintech Group) at room temperature for 60 minutes. Ultrasensitive ECL reagent was subsequently applied for color development. The grayscale values of the images were analyzed using ImageJ software (NIH, USA), and ratios of target protein to internal control protein or phosphorylated protein to corresponding total protein were calculated.

Primary antibodies against JAK1, STAT6, β-actin, and GAPDH were sourced from Proteintech Group (Chicago, USA). The phosphorylated STAT6 (Tyr641) specific antibody was obtained from Affinity Bioscience Co., Ltd (Zhenjiang, China). Antibodies for PI3K and AKT were purchased from Wanlei Bio (Shenyang, China).

### 2.9 Metabolomics study

#### 2.9.1 Sample preparation

The serum samples were rapidly thawed in a water bath at 37°C, followed by vortexing. Subsequently, 100 μL of each sample was then aspirated and placed on ice for 5 minutes. To precipitate proteins, 267 μL of methanol and 133 μL of acetonitrile were added. The mixture was vortexed and oscillated for 10 minutes, then sonicated for another 10 minutes. After sonication, the samples were centrifuged at 12,000 rpm for 10 minutes to separate the supernatant. The supernatant was carefully aspirated and subsequently analyzed by LC-MS.

#### 2.9.2 Chromatographic conditions

The analysis was conducted using a Vanquish Ultra Performance Liquid Chromatograph (Thermo Fisher Scientific, USA) equipped with a Waters ACQUITY UPLC HSS T3 column (2.1 mm × 100 mm, 1.8 μm). Chromatographic separation was achieved with a mobile phase consisted of phase A (5 mmol/L ammonium acetate and 5 mmol/L acetic acid) and phase B (acetonitrile). The gradient elution profile was programmed as follows: 0-0.7 min, flow rate 0.35 mL/min, 99% A; 0.7-9.5 min, flow rate 0.35 mL/min, from 99% to 1% A; 9.5-11.8 min, flow rate from 0.35 mL/min to 0.5 mL/min, 1% A; 11.8-12.0 min, flow rate 0.5 mL/min, from 1% to 99% A; 12.0-14.6 min, flow rate 0.5 mL/min, 99% B. The column and sample tray temperatures were maintained at 40°C and 4°C respectively, with an injection volume of 2 μL.

#### 2.9.3 Mass spectrometry conditions

The analysis was performed using an LTQ Orbitrap Tandem Mass Spectrometer (Thermo Fisher Scientific, USA) with an acquisition time of 0-30 minutes. The mass spectrometer utilized a heated electrospray ionization source (HESI) for both positive and negative ion modes, covering a scanning range of m/z 50-1800. Key settings included an ionization source temperature of 350°C, ionization voltage of 4 kV, capillary voltage of 35 V, and a tube lens voltage of 110 V. Sheath and auxiliary gases, both high-purity nitrogen (> 99.99%), were flowed at rates of 40 and 20 arbitrary units, respectively. Data acquisition employed Fourier transform techniques with a high-resolution full scan, data-dependent DDA-MS2, and mother-ion list PIL-MS2 strategies, complemented by collision-induced dissociation (CID) for mass fragmentation.

#### 2.9.4 Data processing

The chromatographic peaks were aligned and extracted from the plots by Xcalibur software and quantified by Compound Discoverer 3.2 software.

### 2.10 Biosafety evaluation

Following each i.g., the responses and mortality rates of the rats were monitored for 15 consecutive days within a 24-hour period. Serum samples were analyzed for liver function indices (AST, ALT, AST/ALT ratio, ALP, ALB, TP, TBIL) and kidney function indices (UREA, SCR) using a fully automated biochemical analyzer. In addition, liver and kidney tissues from each group were fixed in 4% tissue fixative, dehydrated, embedded in paraffin, and sectioned into 5 μm slices. H&E staining was performed on these sections to identify any histomorphological changes under a light microscope.

### 2.11 Statistical analysis

Statistical analyses and comparisons were conducted using Prism 10 (GraphPad). Data were presented as mean ± standard error of the mean. The normality of data was assessed, and normally distributed datasets were analyzed for statistical significance using one-way ANOVA with Tukey’s multiple comparison test (equal variance), or Welch ANOVA with Dunnett T3 multiple comparisons (unequal variance). Non-normally distributed data were analyzed using the Kruskal-Wallis test with Dunn’s multiple comparison test. Statistical significance was established at p-values < 0.05.

## 3 Results

### 3.1 Characterization of ZRC-CDs

**Figure 2:**
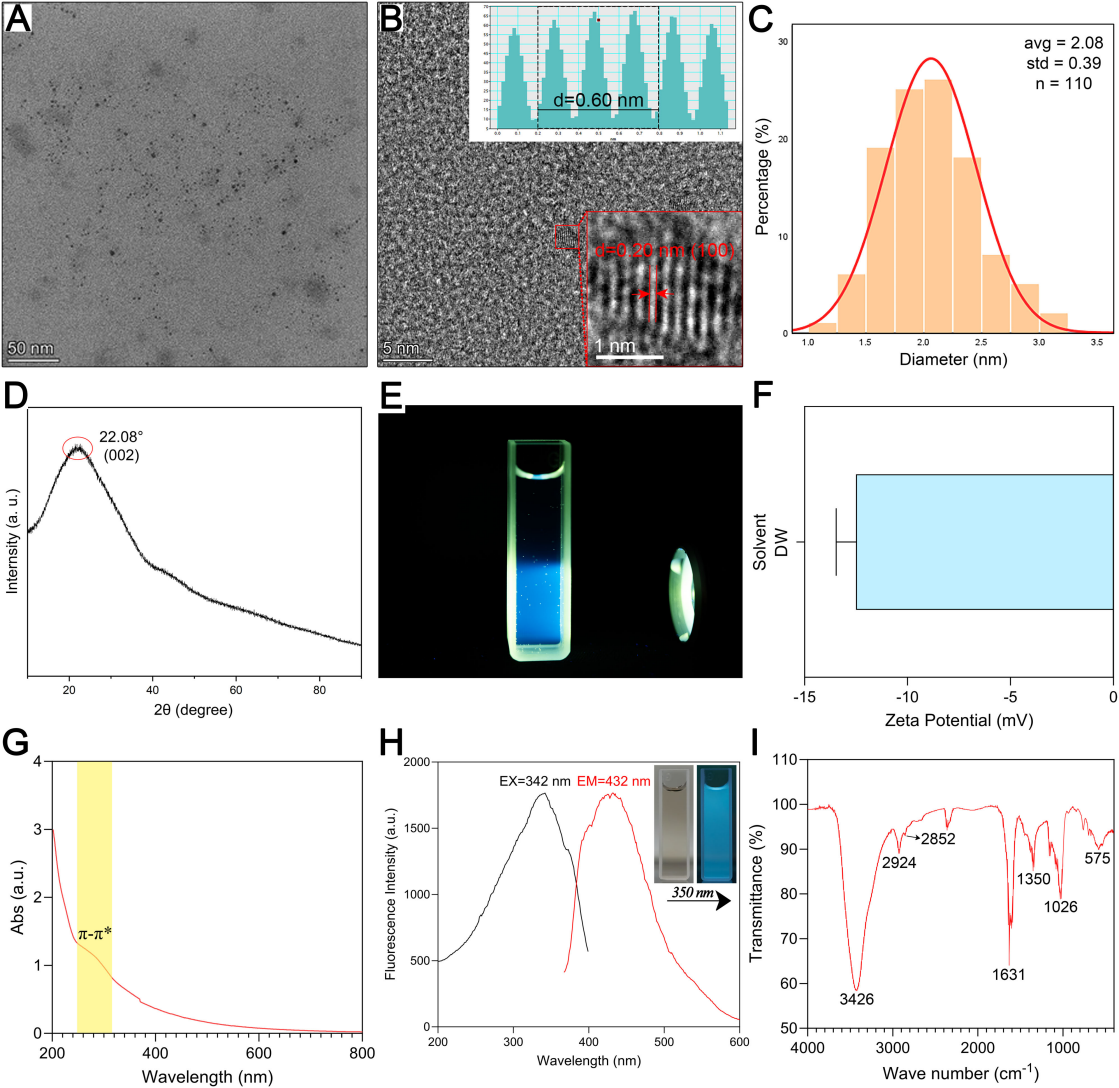
Morphological and optical characterization and component analysis of ZRC-CDs. **(A)** TEM image of ZRC-CDs, scale bars: 50 nm. **(B)** HRTEM image of ZRC-CDs and lattice spacing (indicated by red arrows) of ZRC-CDs (d = 0.20 nm), scale bars: 5 nm and 1 nm, respectively. **(C)** Histogram of particle size distribution. **(D)** XRD pattern of ZRC-CDs. **(E)** The Tyndall effect of ZRC-CDs. **(F)** The zeta potential of ZRC-CDs in DW. **(G)** UV-visible spectrum of ZRC-CDs. **(H)** Fluorescence excitation (black) and emission (red) spectrum of ZRC-CDs. **(I)** FTIR spectrum of ZRC-CDs.

Transmission electron microscopy (TEM) and high-resolution transmission electron microscopy (HRTEM) were employed to analyze the morphology and particle size distribution of ZRC-CDs. TEM images (**Figure 2A**) reveal that ZRC-CDs are spherical with a uniform quasi-spherical structure. The particle size, statistically derived from images of over 110 random nanoparticles, ranged from 1.0 to 3.5 nm (**Figure 2C**). HRTEM images (**Figure 2B**) indicate a lattice spacing of 0.20 nm for the ZRC-CDs, suggesting a structure analogous to the crystalline graphite skeleton, which corresponds to the graphitic carbon (100) crystal plane (32, 33). Furthermore, the X-ray diffraction (XRD) spectrum (**Figure 2D**) displays a broad diffraction peak at 2θ = 22.08°, confirming the (002) plane of graphitic carbon of ZRC-CD (34). This observation aligns with the crystalline graphite skeleton observed in the HRTEM, demonstrating the good crystallinity of ZRC-CDs in different orientations. As shown in **Figure 2E**, the Tyndall effect of the ZRC-CDs solution was obvious, proving its good stability and dispersion. ZRC-CDs have a zeta potential of approximately -12.5 mV, indicating a negative surface charge (**Figure 2F**). Previous studies have shown that negatively charged carbon dots are generally less toxic than positively charged ones (35–37).

The UV-Vis absorption spectra of ZRC-CDs displayed a broad absorption peak at 260 ~ 310nm, which is attributed to the phenomenon of n - π∗ and π - π∗ electron transitions in C=O and C=C bonds (**Figure 2G**) (38, 39). The fluorescence spectra showed the fluorescence characteristics of ZRC-CDs with a maximum emission wavelength of 460 nm and a maximum excitation wavelength of 371 nm (**Figure 2H**).

**Figure 3:**
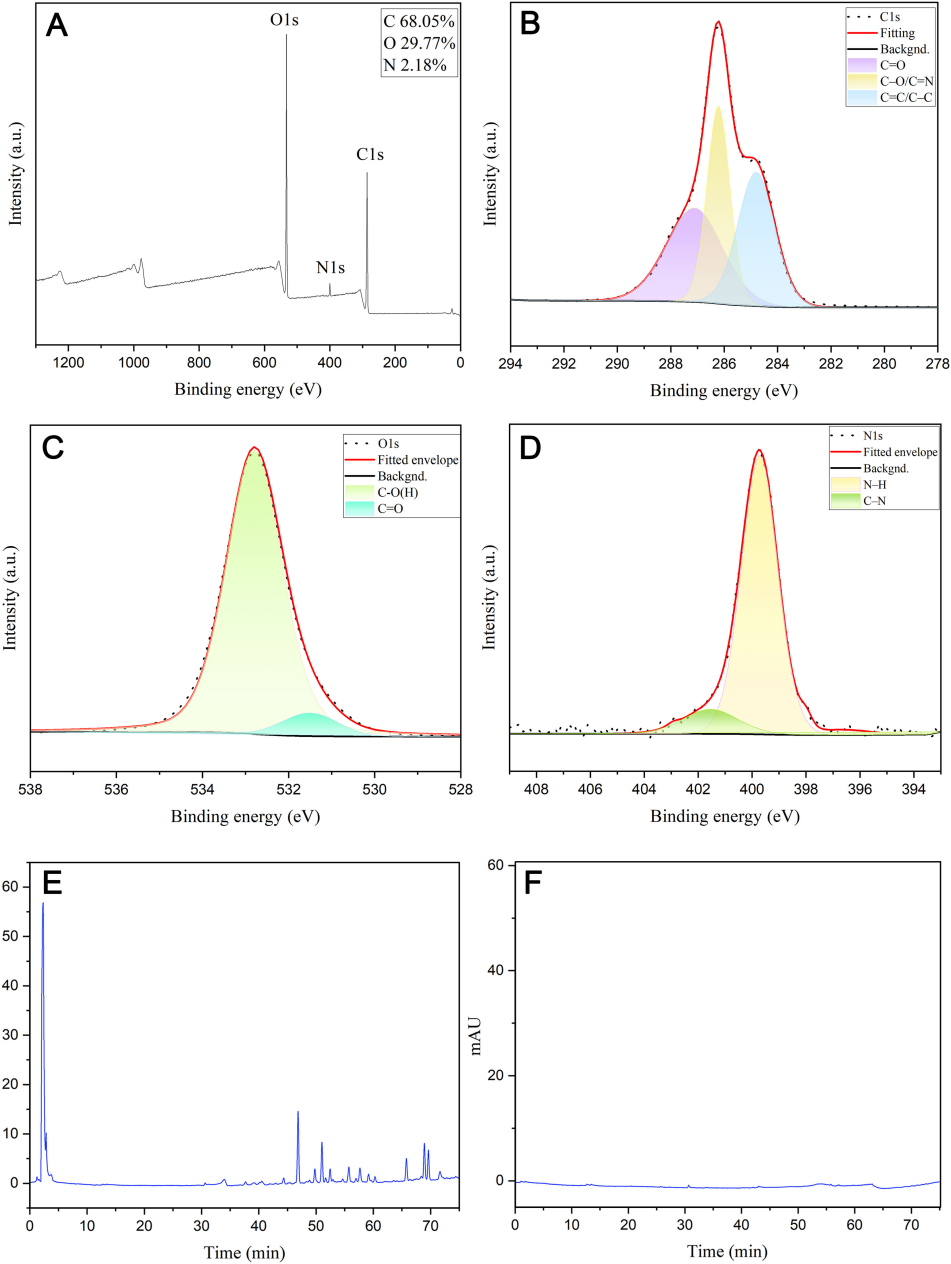
Surface composition and elemental analysis of the prepared ZRC-CDs by XPS and component analysis of ZR and ZRC-CDs by HPLC. **(A)** X-ray photoelectron spectroscopy study of ZRC-CDs. **(B)** C1s, **(C)** O1s, **(D)** N1s. **(E)** HPLC analysis of ZR. **(F)** HPLC analysis of ZRC-CDs.

The FTIR spectra indicated that hydroxyl, carboxyl, and amino functional groups are likely present on the surface of ZRC-CDs (**Figure 2I**). The absorption peak at 3426 cm¹ can be attributed to -OH stretching vibration. The two peaks at 2924 cm¹ and 2852 cm¹ correspond to the asymmetric stretching vibrations of -CH_2_, which are characteristic of methylene groups. In addition, the absorption peak observed at 575 cm¹ corresponds to the in-plane rocking vibration of the -CH_2_ group, further confirming its presence (35). The strong absorption peak at 1631 cm¹ represents the stretching vibration of the C=O bond. Additionally, C-O stretching vibration was observed at 1350 cm¹. The absorption peak at 1026 cm¹ likely indicates C-N stretching vibration (16, 36, 40). Further analysis of ZRC-CDs’ surface composition via XPS identified three predominant elements: C (68.05%), O (29.77%), and N (2.18%) (**Figure 3A**). The high-resolution C1s spectrum (**Figure 3B**) revealed chemical bonds of C-C/C=C at 284.8 eV, C-O/C=N at 286.2 eV, and C=O at 287.1 eV (41, 42). The O1s spectrum indicated peaks at 531.5 eV and 532.8 eV corresponding to C=O and C-O(H) (**Figure 3C**), and the N1s spectrum showed peaks for C-N and N-H at 399.8 eV and 401.6 eV, respectively (**Figure 3D**) (43, 44).

Furthermore, this study analyzed and compared the compositional differences between ZR and ZRC-CDs using high-performance liquid chromatography (HPLC). The ZR chromatogram shows a series of peaks (**Figure 3E**), confirming the presence of small molecule compounds such as flavonoids. In sharp contrast, the chromatographic peaks were almost absent in the methanol extract of ZRC-CDs (**Figure 3F**), indicating that small molecule components were almost absent. After HPLC observation and comparison, the interference of small molecular compounds was eliminated to some extent.

### 3.2 ZRC-CDs alleviate nasal symptoms in OVA-induced AR rats

In the AR rat model, the primary observable nasal allergic symptoms are sneezing and nose scratching. To assess the effect of ZRC-CDs on these symptoms, we recorded the number of sneezes and nose scratches per group over a 10-minute period. After 15 days of nasal drop challenge with OVA solution, the frequency of nose scratching and sneezing significantly increased in the OVA group compared to the control group. In contrast, the frequency of nose scratching and sneezing was significantly reduced in the loratadine, ZRC-CDs-M, and ZRC-CDs-H groups compared to the OVA group (**Figure 1B**). The ZRC-CDs-L group also showed a reduction in the frequency of nose scratching and sneezing compared to the OVA group, although the difference was not statistically significant (**Figure 1C**). These results suggest that ZRC-CDs treatment can alleviate nasal symptoms in AR rats.

### 3.3 ZRC-CDs mitigates histopathological damage in AR rats

The severity of AR is directly correlated with histopathological alterations in the nasal mucosa, assessed further through H&E staining. **Figure 1E**(b) illustrate substantial morphological disruptions in the OVA-induced group, including disruption of cilia continuity, goblet cell proliferation, and increased goblet cell proliferation. In contrast, treatment with ZRC-CDs significantly mitigated these epithelial disturbances, reduced goblet cell proliferation, and attenuated nasal mucosal thickening, as shown in (**Figures 1E, D–F**). These improvements were even more marked in the ZRC-CDs-M group.

### 3.4 ZRC-CDs inhibit OVA-induced type 2 inflammatory response in AR rats

**Figure 4:**
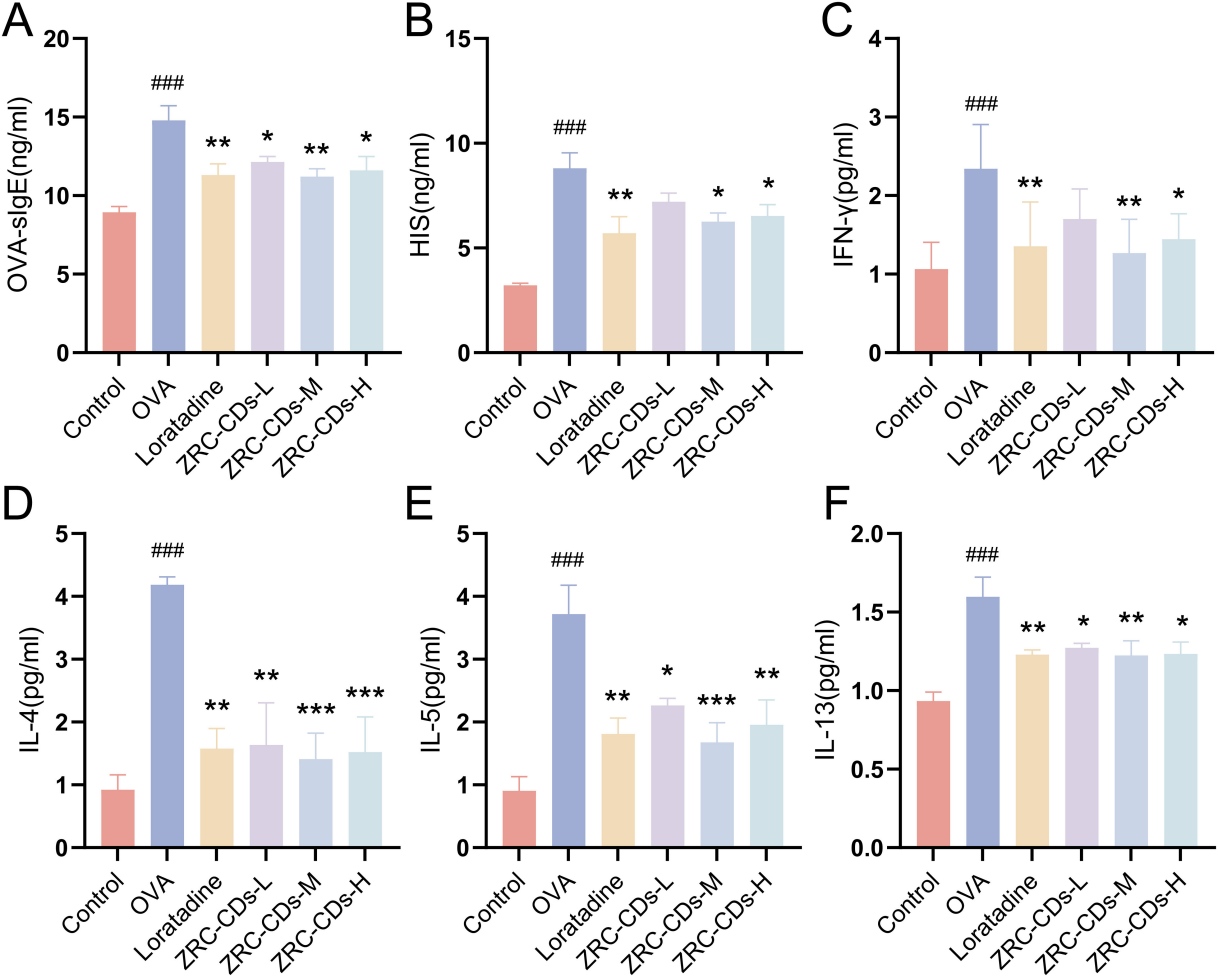
CDs inhibit OVA-induced type 2 inflammatory response and balance Th1/Th2 cytokines in AR rats. Serum levels of **(A)** OVA- specific IgE, **(B)** Histamine, **(C)** IFN-γ, **(D)** IL-4, **(E)** IL-5, **(F)** IL-13 in rats. Each group n = 5. ###*p* < 0.001 vs control group; *p < 0.05, **p < 0.01, ***p < 0.001 vs OVA group.

IL-4, IL-5, and IL-13 are major Th2-type cytokines essential for the development of AR. In contrast, IFN-γ is a typical Th1-type cytokine that inhibits the Th2 response. OVA-sIgE levels specifically indicate OVA sensitization, while histamine is a key inflammatory effector in AR. To assess the efficacy of ZRC-CDs in modulating these biomarkers, we quantified serum levels of inflammatory mediators mentioned above across all study groups. Serum levels of IL-4, IL-5, IL-13, IFN-γ, HIS, and OVA-sIgE were significantly higher in the OVA group compared to the control group, but IFN-γ was the least elevated (**Figures 4A–F**). Compared to the OVA group, serum levels of OVA-sIgE, HIS, IFN-γ, IL-4, IL-5, and IL-13 were significantly reduced in the loratadine, ZRC-CDs-L, ZRC-CDs-M, and ZRC-CDs-H groups to varying degrees, but IFN-γ was the least reduced. These results indicate that ZRC-CDs treatment can reduce the levels of these inflammatory mediators and restores the Th1/Th2 cytokines balance, with the most significant effect observed in the ZRC-CDs-M group, comparable to that of loratadine.

### 3.5 ZRC-CDs restore metabolic abnormalities in AR rats

Recently, metabolomics has increasingly been employed to investigate metabolic changes in inflammatory and allergic diseases, successfully identifying several potential biomarkers and crucial metabolic pathways (45–48). Therefore, we investigated the effect of ZRC-CDs on serum endogenous metabolites. The data were analyzed using SIMCA-P 14.1 software.

**Figure 5:**
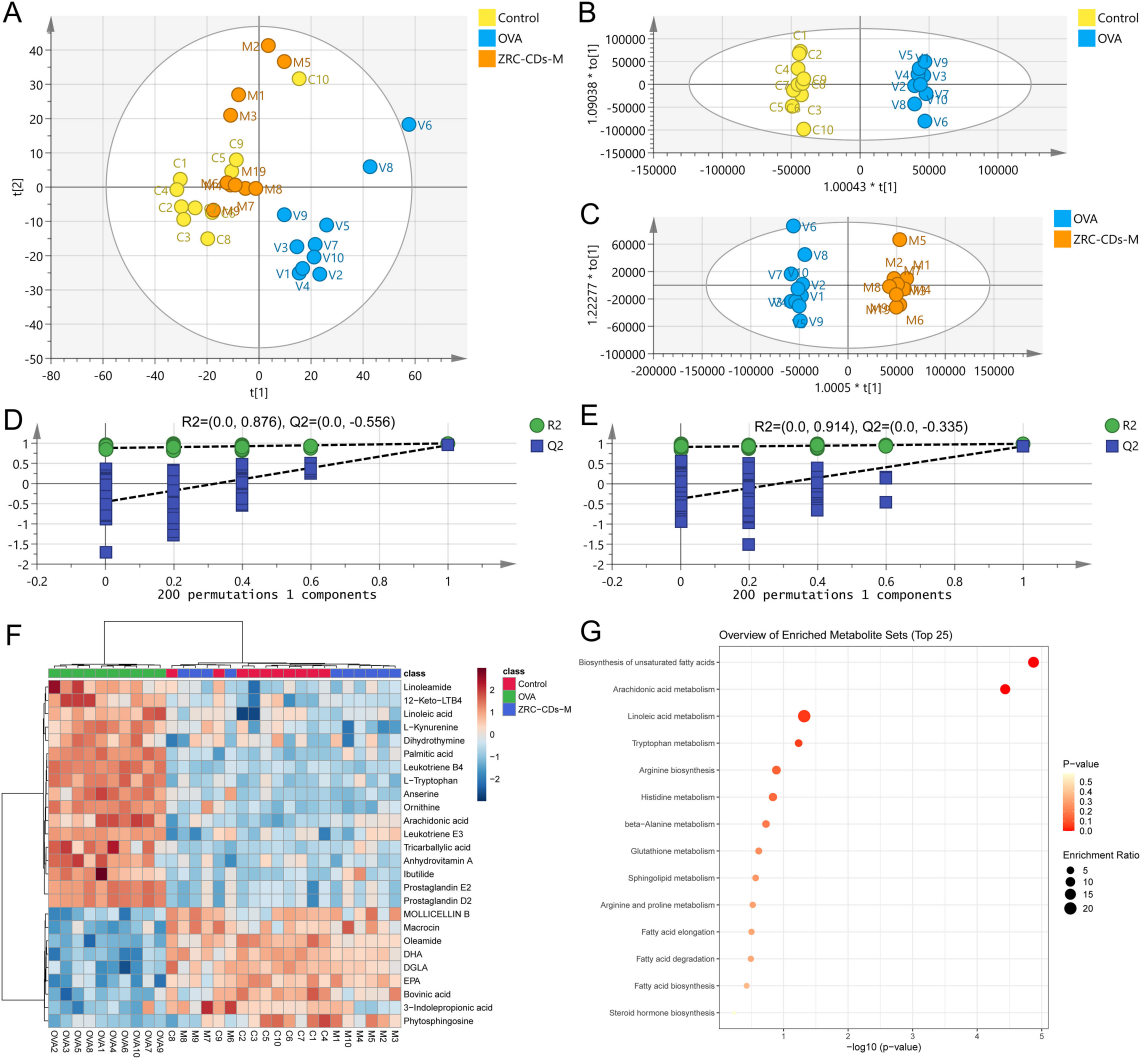
CDs modulated metabolic abnormalities in AR rats. **(A)** PCA score plots of serum metabolic profiling of control, OVA and ZRC-CDs-M groups. OPLS-DA between **(B)** Control and OVA groups and **(C)** their permutation test; **(D)** OPLS-DA between OVA and ZRC-CDs-M groups and **(E)** their permutation test. **(F)** Clustering heatmap of differential metabolites in three groups. **(G)** Enrichment analysis of KEGG metabolic pathway for differential metabolites in three groups. **(H)** Comparison of key differential metabolites in significantly different KEGG metabolic pathways. Each group n = 10.

Principal component analysis (PCA) results (**Figure 5A**) demonstrated that the control, OVA, and ZRC-CDs-M groups were distinctly clustered within each group and clearly separated from each other, indicating marked differences in serum metabolic profiles. Crucially, the ZRC-CDs-M group was closer to the control group than to the OVA group, suggesting that ZRC-CDs could mitigate AR-induced metabolic disturbances.

Orthogonal partial least squares discriminant analysis (OPLS-DA) was employed to eliminate noise unrelated to the categorical information. The results demonstrated distinct separation between the control and OVA groups (**Figure 5B**), as well as between the OVA and ZRC-CDs-M groups (**Figure 5D** To assess model robustness and prevent overfitting, a 200-permutation test was conducted. As shown in (**Figures 5C, E**), the all intercepts of the Q2 regression lines on the y-axis < 0 (C: -0.512, E: -0.324), indicating no overfitting and validating the model’s reliability. Furthermore, by comparing the differences in metabolite levels between the OVA and control groups and between the ZRC-CDs-M and OVA groups, a total of 26 differential metabolites were identified. These were selected based on the criteria of variable importance (VIP) ≥ 1, fold change (FC) > 2, and adjusted *p* < 0.05 (FDR correction).

Differential metabolites were uploaded to the MetaboAnalyst 6.0 database (McGill University, Canada) for further analysis. Clustering heatmaps demonstrated that the levels of metabolites in the ZRC-CDs-M group partially reverted to those of the control group (**Figure 5F**). KEGG enrichment analysis revealed that the protective effects of ZRC-CDs against AR-induced injury in rats were closely related to the regulation of multiple metabolic pathways (**Figure 5G**). These pathways included biosynthesis of unsaturated fatty acids, arachidonic acid metabolism, linoleic acid metabolism, tryptophan metabolism, arginine biosynthesis, histidine metabolism, beta-alanine metabolism, glutathione metabolism, sphingolipid metabolism, arginine and proline metabolism, fatty acid elongation, fatty acid degradation, and fatty acid biosynthesis.

**Figure 6:**
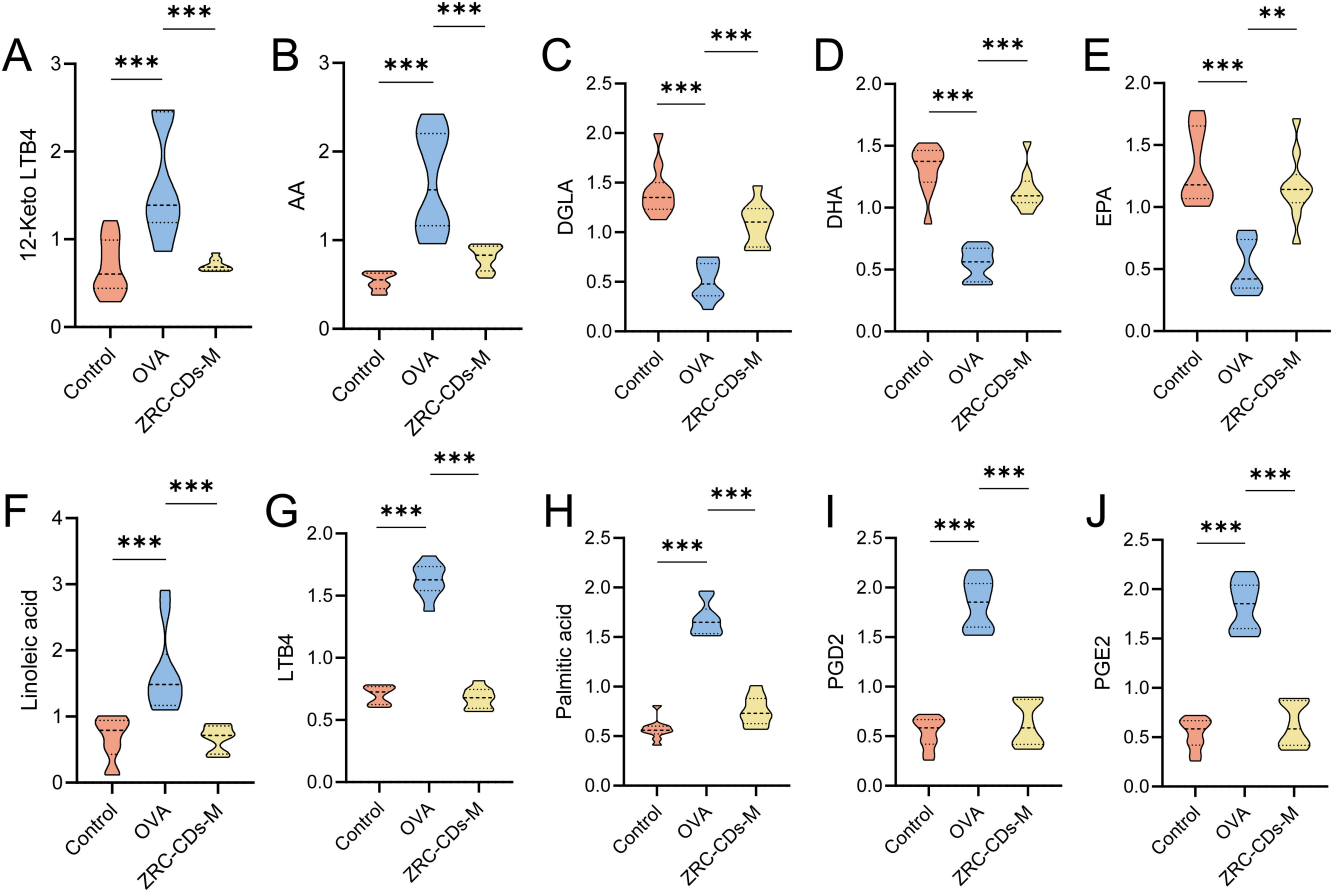
Comparison of key differential metabolites in significantly different KEGG metabolic pathways Among control, OVA, and ZRC-CDs-M Groups **(A–J)**. Each group n = 10. **p < 0.01 and ***p < 0.001 vs control group or OVA group.

Differential metabolites in pathways with *p* < 0.05 (biosynthesis of unsaturated fatty acids and arachidonic acid metabolism) were identified as key differential metabolites. These included arachidonic acid (AA), prostaglandin D2 (PGD2), prostaglandin E2 (PGE2), leukotriene B4 (LTB4), 12-keto-leukotriene B4 (12-keto-LTB4), linoleic acid (LA), palmitic acid (PA), dihomo-gamma-linolenic acid (DGLA), docosahexaenoic acid (DHA), and eicosapentaenoic acid (EPA). In the OVA groups, levels of AA, PGD2, PGE2, LTB4, 12-keto-LTB4, LA, and PA significantly increased, while DGLA, DHA, and EPA significantly decreased compared to the control group (**Figures 6A–J**). These results suggest that OVA induced metabolic pathway disorders in rats, particularly in biosynthesis of unsaturated fatty acid and arachidonic acid metabolism, which were reversed by oral gavage of CDs.

### 3.6 ZRC-CDs inhibit JAK/STAT and PI3K/AKT pathways

The JAK/STAT and PI3K/AKT signaling pathways are pivotal in the pathogenesis of allergic disease. JAK/STAT is highly involved in the differentiation of Th cell subpopulations, while PI3K/AKT increases vascular permeability and affects metabolic pathways, among other things, they are associated with inflammation (49–51). Our research has shown that CDs effectively modulate metabolic disturbances and attenuate its downstream type 2 inflammation in AR rats. To elucidate the mechanisms behind these effects, we investigated the influence of CDs on the JAK/STAT and PI3K/AKT pathways by measuring the protein expression levels of JAK1, p-STAT6, STAT6, PI3K, and AKT in the nasal mucosa of the rats.

**Figure 7:**
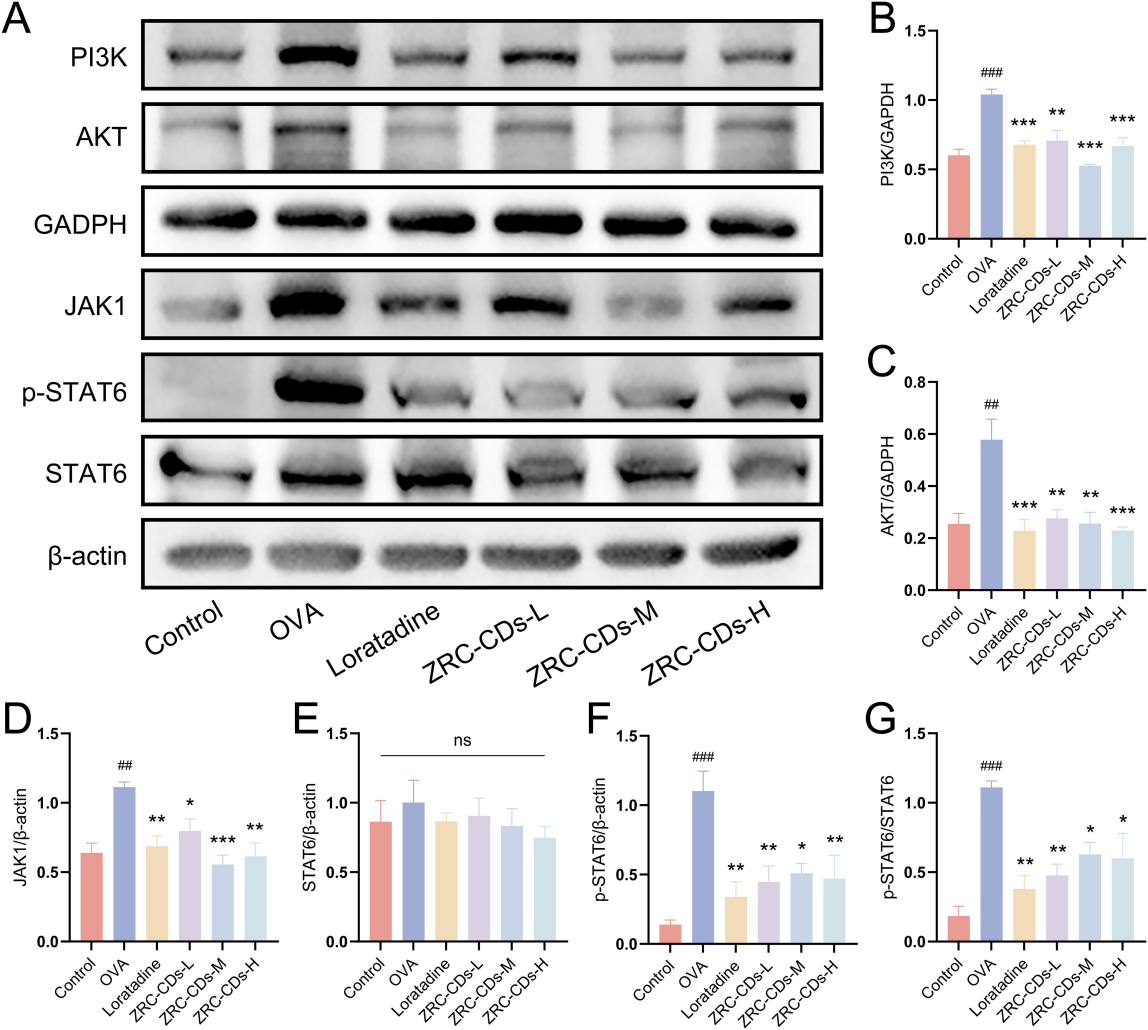
ZRC-CDs inhibits the PI3K/AKT and JAK/STAT signaling pathways in the nasal mucosa. **(A)** Western blot images of PI3K, AKT, JAK1, p-STAT6 and STAT6. **(B–F)** The protein level of PI3K, AKT, JAK1, STAT6 and p-STAT6, respectively. **(G)** the ratio of p-STAT6 to STAT6. Each group n = 3. ##*p* < 0.01, ###*p* < 0.001 vs control group; *p < 0.05, **p < 0.01, ***p < 0.001 vs OVA group; ns means no significant difference between all groups.

Compared to the control group, the expression levels of JAK1, PI3K, and AKT proteins were significantly increased in the nasal mucosa tissues of rats in the OVA group (**Figures 7B, F, G**). There was no significant difference in STAT6 protein levels (**Figure 7C**), but p-STAT6, an activated form of STAT6, was significantly elevated (**Figures 7D, E**). This indicates that OVA intervention resulted in the activation of the JAK/STAT and PI3K/AKT signaling pathways. After treatment with different doses of ZRC-CDs, the levels of JAK1, p-STAT6, PI3K, and AKT proteins in rat nasal mucosa tissues were reduced to varying degrees compared to the OVA group (**Figures 7B–G**). Notably, the ZRC-CDs-M group showed the most significant effect, comparable to that of loratadine. These results confirm that the JAK/STAT and PI3K/AKT signaling pathways are involved in the development of AR and that ZRC-CDs can inhibit the activation of these pathways to some extent.

### 3.7 Biosafety assessment

**Figure 8:**
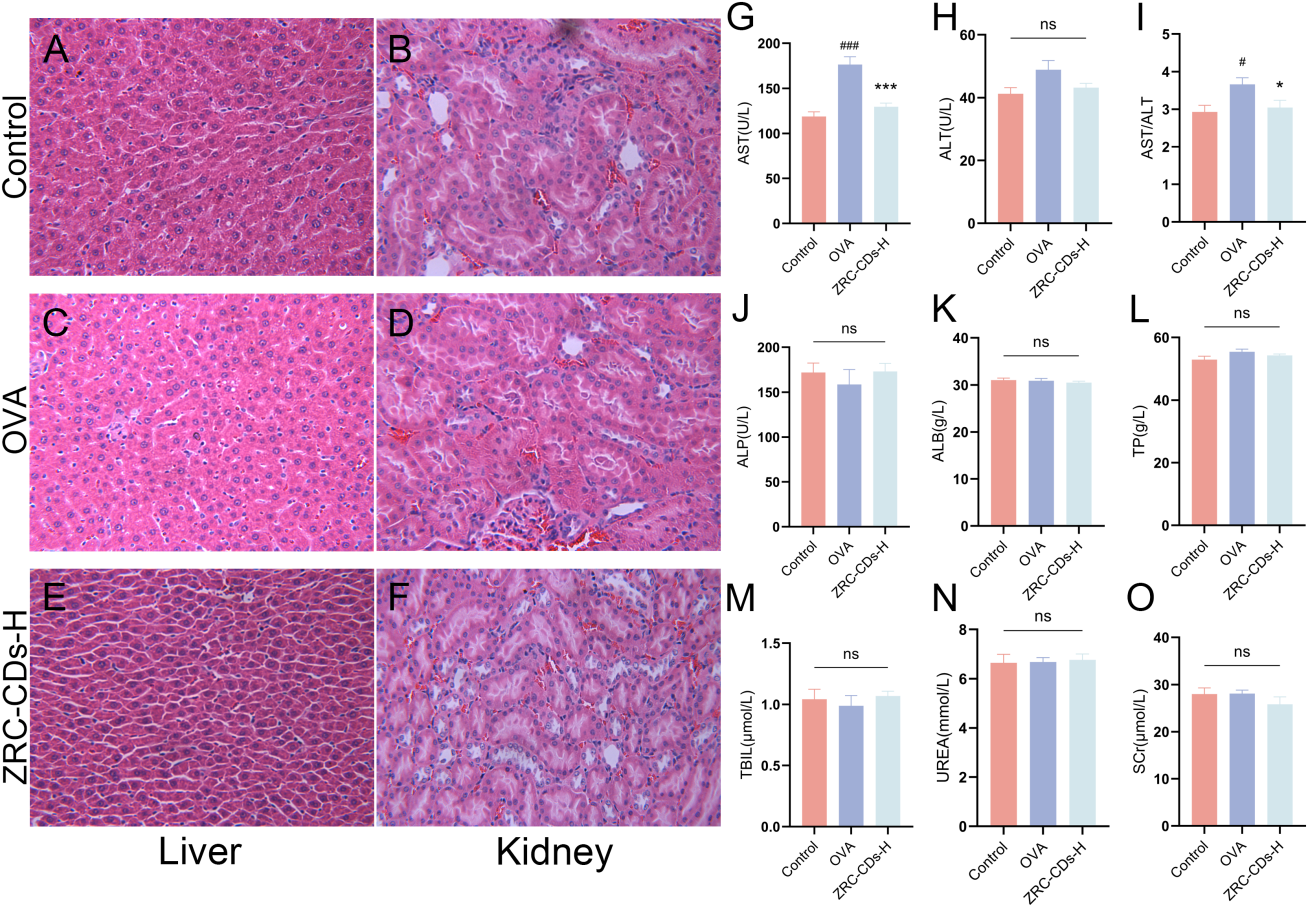
Biocompatibility and biotoxicity analysis of ZRC-CDs. H&E stained images of **(A, C, E)** liver and **(B, D, F)** kidney pathological sections from control, OVA and ZRC-CDs-H groups. Scale bar: 10x. Serum **(G–M)** Liver and **(N, O)** kidney function parameters of control, OVA and ZRC-CDs-H groups. Each group n = 10. #*p* < 0.05 and ###*p* < 0.001 vs control group; *p < 0.05 and ***p < 0.001 vs OVA group; ns means no significant difference between all groups.

Given that CDs are primarily excreted through the liver and kidneys, we examined the potential toxicity of ZRC-CDs by measuring liver and kidney function parameters and performing H&E staining of liver and kidney tissues (52). Throughout the experimental timeline, no rats died before blood samples and organs were collected, which was consistent with the control group. As shown in **Figures 8A-F**, H&E staining of liver and kidney revealed no significant histological changes after the ingestion of high-dose ZRC-CDs solution compared to the control and OVA groups. Additionally, OVA did not cause significant changes in liver and kidney function parameters, except for an increase in AST, but ZRC-CDs mitigated these damages (**Figures 8G–O**). These results suggest that the *in vivo* toxicity of ZRC-CDs is low, corroborating previous *in vitro* results (35, 53–55).

## 4 Discussion

Carbon dots are increasingly employed across biomedical, sensing, and optoelectronic fields owing to their superior photostability, high biocompatibility, and excellent water solubility (56–58). In the biomedical field, although the increasing adoption of CDs in disease diagnostics and drug delivery systems, research into their inherent biological activities is still nascent. This gap in knowledge constrains the full exploitation of their potential. Remarkably, CDs derived from herbal medicine naturally possess multifunctional groups and inherent biological activities, thereby streamlining their application in therapeutic contexts (59–62). CDs derived from *Phellodendri Chinensis Cortex* have shown marked improvement in the appearance and severity indices of psoriatic lesions in mouse models, alongside demonstrable immunomodulatory effects on macrophages (17). Additionally, Qiang et al. reported that CDs derived from *Carthami Flos* and *Angelicae Sinensis Radix* not only reduced joint friction in rheumatoid arthritis rat models but also exhibited significant anti-inflammatory properties (60). These findings underscore the substantial bioactivity and therapeutic potential of carbon dots. Additionally, their production is environmentally benign, low toxic, simple, and cost-efficient, aligning with the principles of green chemistry and the growing interest in biomass-derived carbon dots (21, 23–25).

Inspired by Traditional Chinese Medicine, where charcoal herbal medicines are prepared through a process akin to the high-temperature pyrolysis method used for CDs, and given the clinical efficacy of ZRC in treating respiratory diseases such as AR and asthma, we hypothesized that ZRC contains CDs produced by the charcoal process and capable of alleviating AR.

Therefore, we extracted and purified CDs from ZRC and then found that its particle size distribution was in the range of 1.0-3.5 nm and had good water dispersibility. ZRC-CDs was confirmed by FTIR and XPS spectroscopy to be rich in surface functional groups, which are likely one of the contributors to their unique bioactivity, good water dispersibility and excellent biocompatibility. Subsequently, an AR model was induced in rats using OVA. Behavioral assessments indicated that oral gavage of CDs mitigated the clinical symptoms of AR. Furthermore, histopathological analysis via H&E staining of the nasal mucosa demonstrated that CDs significantly reduced the pathological damage associated with AR.

The pathophysiology of AR is multifaceted, encompassing environmental, genetic, and immunological contributors, with the predominant mechanism being a Type 2 immune response (49). Upon exposure to allergens, epithelial cells trigger dendritic cells (DCs) through the secretion of alarmins (IL-33, IL-25, and TSLP), prompting the transformation of naive T-cells into Th2 cells. These Th2 cells secrete IL-4 and IL-13 to facilitate IgE class switching and IL-5 to recruit eosinophils. Additionally, IL-13 promotes mucus production by goblet cells. Subsequently, IgE activates mast cells to release inflammatory mediators like histamine, leukotrienes, and prostaglandins, leading to the typical symptoms of nasal inflammation (1, 63). Furthermore, recent findings indicate that innate lymphoid cells type 2 (ILC2) also emit Th2 cytokines upon activation by epithelial-derived alarmins (64). Our analysis of IL-4, IL-5, IL-13, IFN-γ, OVA-sIgE, and histamine levels revealed that CDs effectively suppress this inflammatory type 2 immune response and restores the Th1/Th2 cytokines balance.

Lipid metabolism plays a crucial role in the human body, and previous studies have shown that lipid metabolic homeostasis is significantly disrupted in AR (48, 65, 66). Similarly, our metabolomic analysis identified significant alterations in metabolic pathways and metabolite levels in AR-induced rats. Notably, CDs were shown to modulate these changes, significantly affecting 2 distinct metabolic pathways and altering 10 key specific metabolites.

AA metabolism plays a pivotal role in inflammatory processes. Linoleic acid, a precursor in AA metabolism, undergoes conversion to DGLA and subsequently to AA via a two-step enzymatic process. AA is then transformed into pro-inflammatory eicosanoids, such as PGD2 and LTB4, via the cyclooxygenase and lipoxygenase pathways (51). PGD2 and LTB4 not only activate DCs but also promote Th2 cell proliferation, eosinophil recruitment, and amplify inflammatory responses (67–70). Conversely, EPA and DHA generate anti-inflammatory eicosanoids that counteract the effects of AA metabolites (71–73). In addition to this, phytosphingosine, kynurenine, tryptophan, ornithine, and oleamide also underwent significant changes in allergic diseases, indicating potential therapeutic or pathological roles in AR (**Supplementary Table 1**), which was also confirmed by relevant studies (47, 74–78).

To understand how ZRC-CDs modulate metabolism and downstream inflammation, we focused on key upstream proteins within critical signaling pathways. The JAK/STAT signaling pathway regulates inflammatory Th2 cell responses, with STAT6 responding to IL-4 and IL-13 via JAK1 phosphorylation (79, 80). JAK also activates PI3K, which, along with its downstream effector Akt, plays a crucial role in regulating cellular metabolism. Moreover, the PGD2 receptor (DP2) can also indirectly activate the PI3K pathway (81). This crosstalk supports the findings of our study, highlighting a significant interplay between these signaling molecules. Our study confirmed that the JAK/STAT and PI3K/AKT signaling pathways, which are crucial in AR pathogenesis, were activated during AR episodes. Importantly, these pathways were notably inhibited following oral administration of ZRC-CDs. This modulation suggests that ZRC-CDs could serve a therapeutic function in AR by influencing lipid metabolism and type 2 inflammatory responses through these pathways.

As CDs have rapidly advanced, their potential toxicity has also come under scrutiny. This study demonstrated that ZRC-CDs exhibit low *in vivo* toxicity, as evidenced by liver and kidney function tests and histopathological evaluations. Notably, OVA caused an elevation of AST in AR rats, indicating potential mild damage to hepatocytes or cardiomyocytes, which was mitigated by CDs. This exceptional biocompatibility positions CDs as promising agents for AR treatment. Meanwhile, its cardiomyocytoprotective or hepatocytoprotective effects can be further explored in the future.

Although this study has shown that ZRC-CDs regulate lipid metabolism and type 2 inflammation at the molecular level in AR, the *in vivo* distribution and precise mechanisms remain elusive due to the complex interactions of CDs upon entry into the organism. Future research should focus on three primary areas: more comprehensive characterization to recognize CDs; quantification of immune cells to identify cell populations targeted by CDs and to elucidate their underlying cellular mechanisms of action; enhance molecular level understanding of these mechanisms using multi-omics techniques and perform targeted knockout or rescue experiments to validate these findings and identify precise triggers for AR mitigation by ZRC-CDs.

## 5 Conclusion

In conclusion, our research identified CDs derived from ZRC, which inherently possess multifunctional groups and unique bioactivities without requiring additional surface modifications. We established an OVA-induced AR rat model and observed that ZRC-CDs effectively alleviated nasal symptoms and pathological damage in AR rats. In addition, ZRC-CDs modulated lipid metabolism and type 2 inflammatory response, and inhibited PI3K/AKT and JAK/STAT pathways associated with metabolism and inflammation. Notably, the CDs displayed superior biocompatibility, underscoring their promise as an anti-allergic rhinitis drug candidate. Our findings not only offer new therapeutic avenues for treating allergic rhinitis but also enhance understanding of the bioactivities associated with herbal nanomedicine, thereby facilitating broader clinical applications and acceptance of ZRC. This dual contribution not only helps to expand the biomedical uses of CDs, but also helps to promote wider clinical applications of ZRC.

## Data Availability

The original contributions presented in the study are included in the article/**Supplementary Material**. Further inquiries can be directed to the corresponding authors.

## Supplementary material

Supplementary Figure 1 Abstract graphics.
