# Supplementary figures and images for: Green carbon dots derived from Zingiberis Rhizoma Carbonisatum alleviate ovalbumin-induced allergic rhinitis
Source: Front Immunol. 2024 Nov 28;15:1492181. doi: 10.3389/fimmu.2024.1492181 (PMC11634691; doi:10.3389/fimmu.2024.1492181)

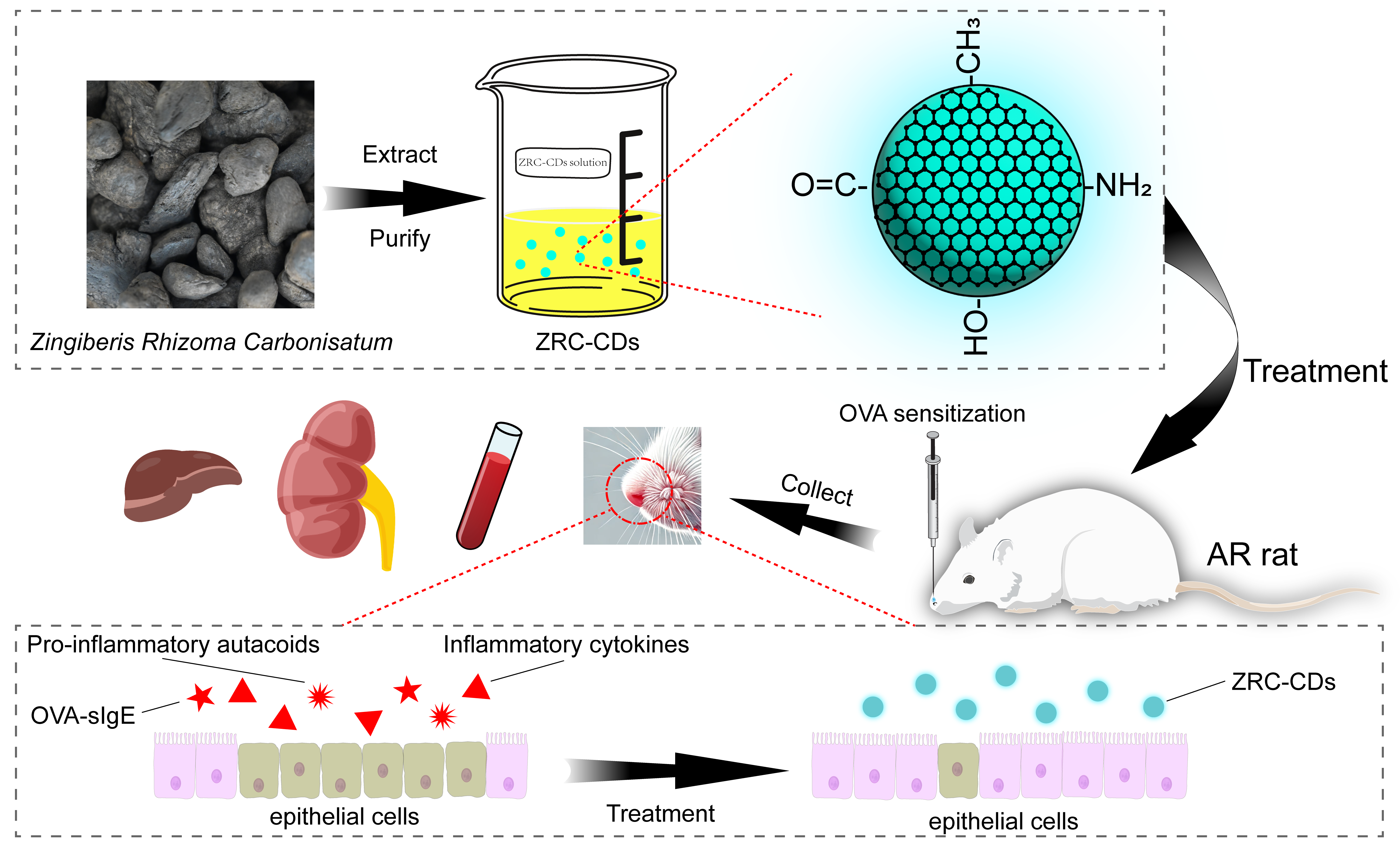

Supplement: Supplementary Figure 1 — Abstract graphics. [file Image1.tif]
